# Supplementary material for: The safety and efficacy of phosphodiesterase type 5 inhibitors in the treatment of diabetic erectile dysfunction: a systematic review and meta-analysis
Source: PeerJ. 2025 Oct 7;13:e20147. doi: 10.7717/peerj.20147 (PMC12513374; doi:10.7717/peerj.20147)
Supplement: Supplemental Information 1 [file peerj-13-20147-s001.docx]

This study aims to provide clinicians and researchers in the field of endocrinology and andrology with evidence-based evidence and treatment recommendations for diabetic erectile dysfunction, so as to help them make better clinical decisions and research.
